# Microsurgical Onco‐Testicular Sperm Extraction in a Patient With Testicular Cancer and Congenital Unilateral Testicular Absence: A Case Report

**DOI:** 10.1002/iju5.70191

**Published:** 2026-05-10

**Authors:** Yuki Matsumoto, Toshiyasu Amano, Mamoru Hachiya, Tomohisa Fujita, Tetsuya Imao

**Affiliations:** ^1^ Department of Urology Nagano Red Cross Hospital Nagano Japan; ^2^ Center for Reproductive Medicine Nagano Red Cross Hospital Nagano Japan

**Keywords:** azoospermia, congenital abnormalities, fertility preservation, testicular neoplasms, testicular sperm extraction

## Abstract

**Introduction:**

Testicular cancer primarily affects men of reproductive age. Therefore, fertility preservation is important in managing testicular cancer. In patients with a solitary testis, oncological testicular sperm extraction (onco‐TESE) is used to retrieve sperm from tumor‐bearing testis.

**Case Presentation:**

A 39‐year‐old man with congenital absence of the left testis presented with a 4‐month history of progressive right testicular swelling. Laboratory tests showed elevated β‐human chorionic gonadotropin and follicle‐stimulating hormone concentrations, and ultrasonography showed a heterogeneous hypoechoic mass with microcalcifications. A seminal analysis showed azoospermia with AZFc partial deletion. Following orchiectomy, microsurgical onco‐TESE was performed. Motile sperm were successfully retrieved from preserved seminiferous tubules. A pathological examination demonstrated a pure seminoma. In preserved seminiferous tubules, the Johnsen score was 9–10, and partial tubular hyalinization and microcalcifications were observed.

**Conclusion:**

Microsurgical onco‐TESE is for retrieving sperm in patients with testicular cancer with a solitary testis, even in the presence of impaired spermatogenesis.

## Keynote Message

- Testicular cancer developing in congenital unilateral testicular absence is extremely rare, and fertility preservation at the start of treatment is important.
- Onco‐TESE is a useful fertility preservation treatment for patients with testicular cancer who have bilateral tumors or unilateral testicular absence.
- A tumor‐bearing testis may have complicated spermatogenic dysfunction; therefore, microscopic onco‐TESE is a useful method for patients with azoospermia and testicular cancer.

**Abbreviations**
FSH: follicle‐stimulating hormone
Onco‐TESE: oncological testicular sperm extraction

## 1 Introduction

Testicular cancer predominantly affects young men of reproductive age, and fertility preservation is an important issue in its management. The initial treatment for testicular cancer is radical orchiectomy. However, in patients with a solitary testis, oncological testicular sperm extraction (onco‐TESE) is an effective option for fertility preservation by retrieving sperm from the tumor‐bearing testis. We report a rare case of azoospermia with right testicular cancer and congenital absence of the left testis, in which microsurgical onco‐TESE was successful despite suspected impaired spermatogenesis.

## 2 Case Presentation

A 39‐year‐old man with congenital absence of the left testis presented to a local urology clinic with a 4‐month history of progressive right testicular swelling. He had married 4 years earlier and had a 39‐year‐old wife, but they had no children.

He was diagnosed with a right testicular tumor and referred to our hospital for male infertility treatment with fertility preservation.

**FIGURE 1:**
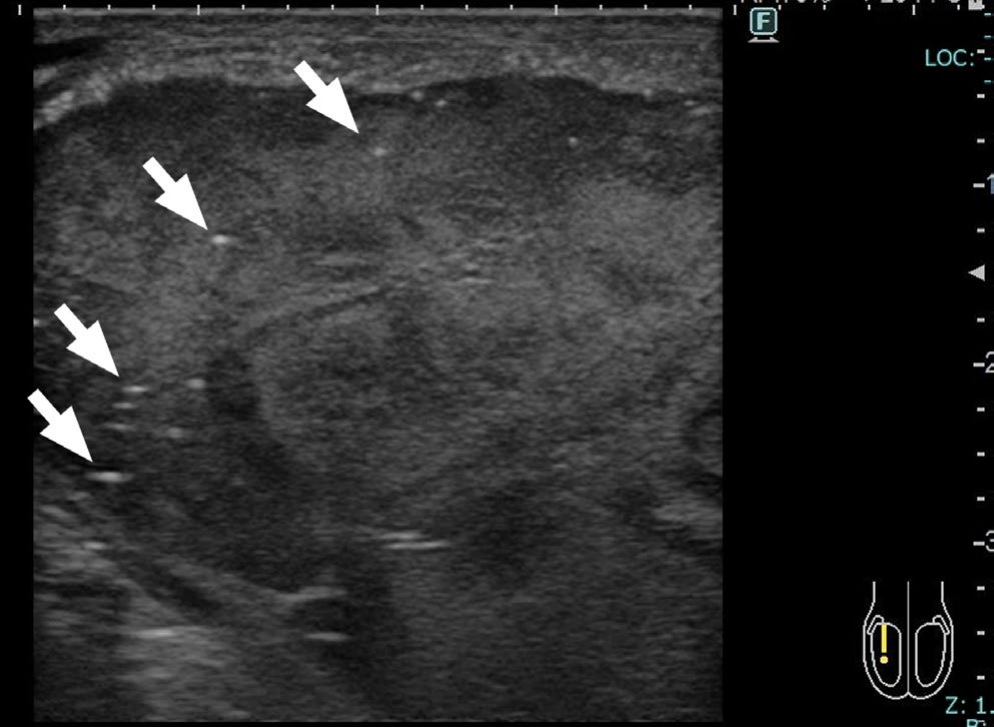
Ultrasonographic findings of the right testis. The tumor occupies most of the testis. The tumor shows heterogeneous echogenicity and contains numerous hyperechoic foci (arrows).

On a physical examination, the right testis was hard and markedly enlarged to approximately the size of a large hen's egg, and there was no thickening of the right spermatic cord. The left testis was non‐palpable. Serum tumor markers showed an elevated β‐human chorionic gonadotropin concentration of 12.2 mIU/mL. Endocrinological tests showed an elevated luteinizing hormone concentration of 15.5 mIU/mL and follicle‐stimulating hormone (FSH) concentration of 84.8 mIU/mL, with a normal testosterone concentration. Scrotal ultrasonography demonstrated a diffusely heterogeneous hypoechoic right testis with focal microcalcifications (Figure 1). Contrast‐enhanced computed tomography showed no distant metastases. A semen analysis showed azoospermia. The karyotype was 46, XY, and Y‐chromosome microdeletion testing identified a partial deletion in the AZFc region.

**FIGURE 2:**
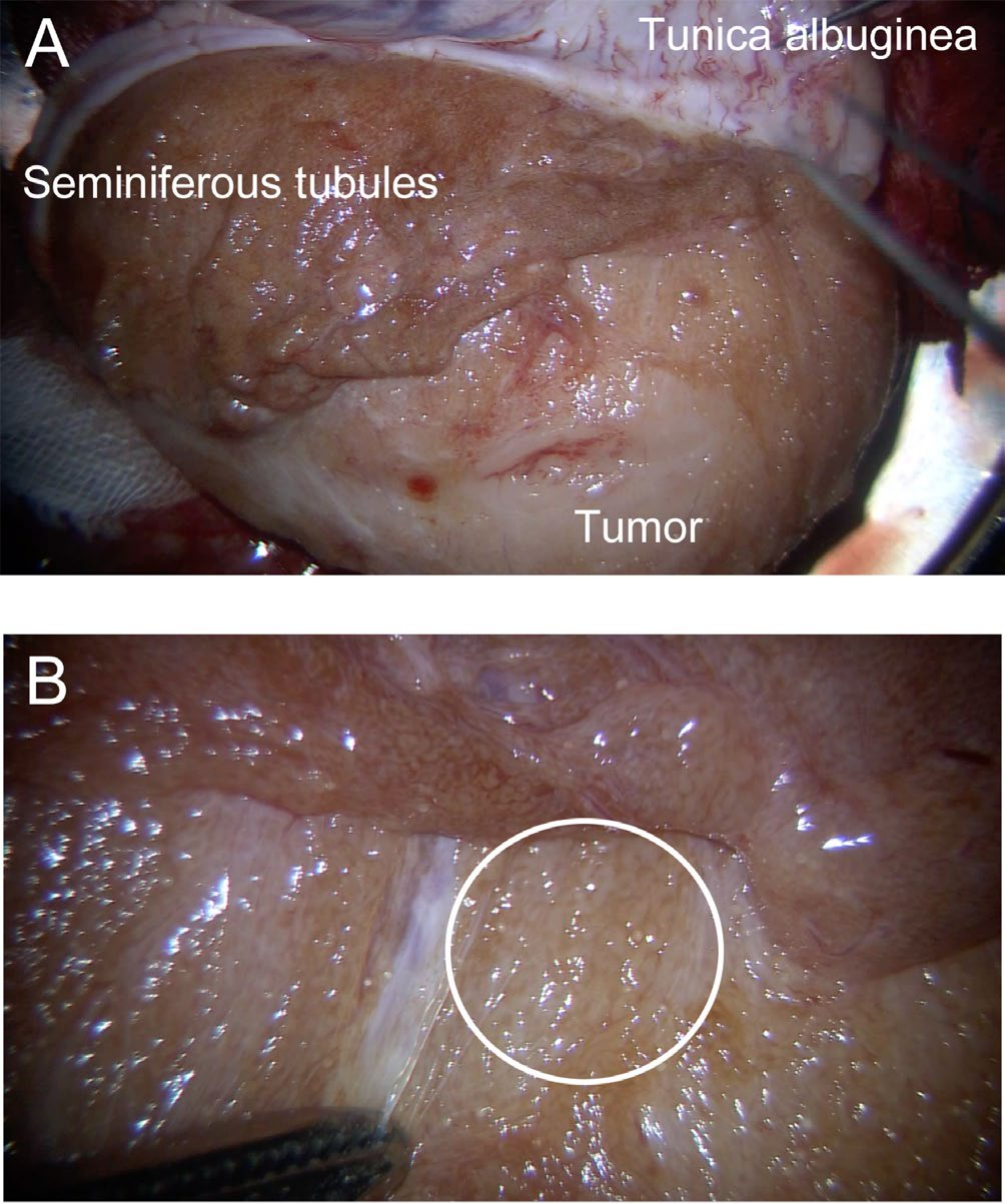
Surgical findings during microdissection TESE. (A) Macroscopic findings after incision of the tunica albuginea. The seminiferous tubules can be seen immediately beneath the tunica albuginea, compressed by the tumor. (B) Microscopic findings. Partially thickened and opaque seminiferous tubules can be seen (circle).

A right radical inguinal orchiectomy was performed for a testicular tumor and azoospermia, followed by microdissection TESE on the excised testis. Macroscopically normal seminiferous tubules compressed by the tumor were identified just beneath the tunica (Figure 2A). Partially thickened and opaque seminiferous tubules were observed with an operation microscope unit (Mitaka Kohki, Mitaka, Japan) (Figure 2B). These areas were sampled, and motile sperm were successfully retrieved. The operative time was 40 min, and a total of five tubes of sperm were successfully cryopreserved. Each tube contains at least approximately 500 spermatozoa.

**FIGURE 3:**
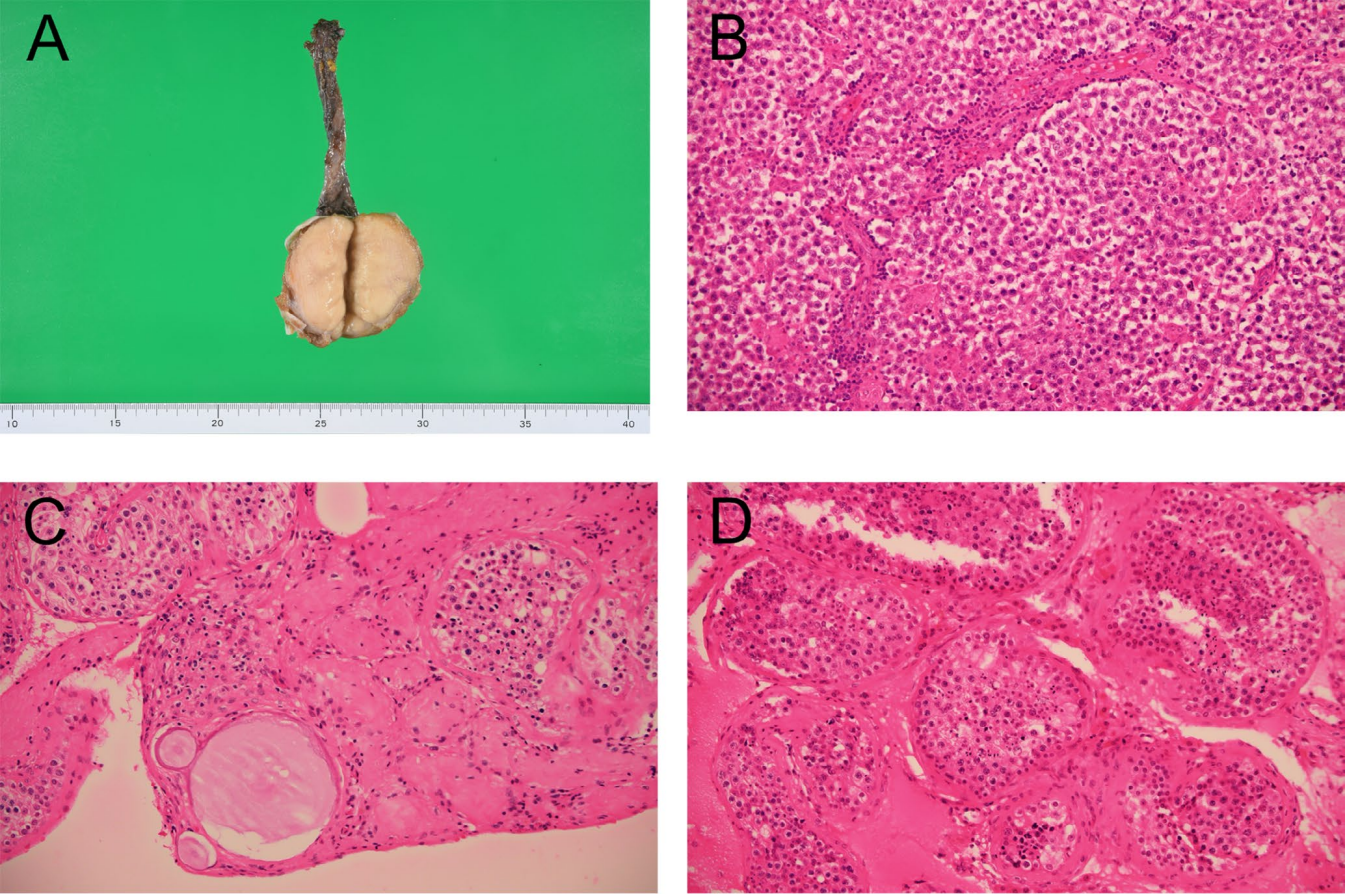
Resected specimen and pathological findings. (A) The testis was largely replaced by the tumor. (B) Hematoxylin–eosin staining of a tumor section (original magnification, ×200). The tumor was a pure seminoma. (C and D) Hematoxylin–eosin staining of the seminiferous tubule region (original magnification, ×200). (C) Microcalcifications are present within the tubules, and hyalinized tubules can be seen surrounding them. (D) In normal seminiferous tubules, the Johnsen score was 9–10.

In the excised specimen, the testis was largely replaced by the tumor, with normal tissue visible only marginally at the tumor periphery (Figure 3A). In hematoxylin–eosin staining, the tumor consisted of a pure seminoma (Figure 3B). Germ cell neoplasia in situ was present in some seminiferous tubules. In a section of normal tissue, microcalcifications were observed within the seminiferous tubules, and hyalinized seminiferous tubules were present around them (Figure 3C). No spermatozoa were observed in the hyalinized tubules. In another section of normal tissue, spermatozoa were observed with a Johnsen score of 9–10 (Figure 3D).

## 3 Discussion

Testicular cancer typically occurs in reproductive men aged 15–40 years [1, 2]. Therefore, fertility preservation is crucial in the treatment of testicular cancer. In cases of testicular cancer affecting one side, sperm cryopreservation is often performed before chemotherapy because the contralateral testis is usually normal. However, in a tumor‐bearing testis, sperm need to be retrieved in cases of azoospermia, bilateral testicular tumors, or testicular cancer occurring in a solitary testis.

Bilateral testicular tumors occur in approximately 0.5%–5% of testicular cancer cases. Although these tumors are often metachronous, 20%–35% are synchronous [3, 4]. Congenital testicular absence is often detected during evaluation for cryptorchidism, and 5%–20% of cryptorchid cases are ultimately diagnosed as testicular absence [5]. The causes of congenital testicular absence are thought to include thrombosis, torsion of the testicular vessels before or at birth, or endocrine disorders [6]. While cryptorchidism is a risk factor for testicular cancer, unilateral congenital testicular absence is not considered a risk factor for contralateral testicular cancer [5]. Therefore, the estimated frequency of testicular cancer in patients with congenital testicular absence is approximately 0.05%–0.2%. Although several reports have described onco‐TESE in testicular cancer, most involved synchronous or metachronous bilateral disease [7, 8]. To the best of our knowledge, onco‐TESE performed in patients with congenital testicular absence is extremely rare [9].

There are two types of onco‐TESE: “narrowly defined onco‐TESE,” in which sperm is retrieved from removed testicles with tumors, as in this case; and “broadly defined onco‐TESE,” in which TESE is performed prior to cancer treatment regardless of the type of cancer [10]. In this report, “narrowly defined onco‐TESE” is referred to as onco‐TESE. In Japan, there have been several reports on onco‐TESE [10, 11]. However, we are afraid that not all cases of onco‐TESE have been reported; thus, the frequency of onco‐TESE procedures remains unclear. Generally, onco‐TESE is performed immediately after orchiectomy [12]. There are no reports specifying a time limitation between testicular removal and sperm retrieval. However, since prolonged ischemia leads to a decline in sperm quality, sperm retrieval should be performed as soon as possible after removal. Furthermore, there have been no reports of cancer cell contamination. This is a safe procedure that can be performed by distinguishing between the tumor and normal seminiferous tubules under direct visualization or microscopic observation [12].

The reported sperm retrieval rate of onco‐TESE in testicular cancer ranges from 40% to 70% [7, 10, 11, 12, 13, 14]. Conventionally, tissue is sampled from macroscopically tumor‐free parenchyma; however, microsurgical onco‐TESE has recently been described [12, 14, 15]. The association between testicular cancer and impaired spermatogenesis is well recognized [16, 17]. Therefore, microsurgical extraction is particularly beneficial in patients with severe spermatogenic dysfunction. In this case, spermatogenic dysfunction was suspected due to marked elevation of FSH and partial deletion of AZFc. Using microsurgical onco‐TESE, we were able to efficiently retrieve motile sperm.

Microcalcification was observed in the preserved normal testicular region in this case. Testicular microlithiasis is characterized by the presence of multiple hyperechoic foci without acoustic shadowing within the testis on ultrasonography. Pathologically, laminated calcifications are observed within the seminiferous tubules. Testicular microlithiasis is thought to result from calcification of degenerated germ cells or sloughed epithelial remnants [18]. Although the clinical significance of testicular microlithiasis remains controversial, associations between testicular microlithiasis and testicular cancer and infertility have been suggested [19, 20]. In this case, the presence of multiple microcalcifications and hyalinized seminiferous tubules in the tissue around the tumor was considered findings that support such associations.

## 4 Conclusion

Onco‐TESE is an effective fertility preservation method for patients with testicular cancer. Even in cases of azoospermia presenting with findings suggestive of severe spermatogenic dysfunction, such as a marked elevation in FSH or partial deletion of the AZFc region, microsurgical onco‐TESE is useful and should be considered.

## Consent

Informed consent was obtained from the patient.

## Conflicts of Interest

The authors declare no conflicts of interest.

## Data Availability

The data that support the findings of this study are available from the corresponding author upon reasonable request.

## References

[1] J. K. Gurney , A. A. Florio , A. Znaor , et al., “International Trends in the Incidence of Testicular Cancer: Lessons From 35 Years and 41 Countries,” European Urology 76 (2019): 615–623. 31324498 10.1016/j.eururo.2019.07.002 PMC8653517

[2] N. Singla , A. Bagrodia , E. Baraban , C. D. Fankhauser , and Y. M. A. Ged , “Testicular Germ Cell Tumors: A Review,” Journal of the American Medical Association 333 (2025): 793–803. 39899286 10.1001/jama.2024.27122

[3] K. P. Dieckmann , P. Anheuser , F. Sattler , T. Von Kügelgen , C. Matthies , and C. Ruf , “Sequential Bilateral Testicular Tumours Presenting With Intervals of 20 Years and More,” BMC Urology 13 (2013): 71. 24321309 10.1186/1471-2490-13-71 PMC4028980

[4] T. Amano , T. Imao , and K. Takemae , “Male Infertility and Androgen Replacement Therapy for Subjects With Bilateral Testicular Tumors,” Reproductive Medicine and Biology 13 (2014): 103–106. 29699154 10.1007/s12522-013-0171-z PMC5906938

[5] Ö. Pirgon and B. N. Dündar , “Vanishing Testes: A Literature Review,” Journal of Clinical Research in Pediatric Endocrinology 4 (2012): 116–120. 22985611 10.4274/Jcrpe.728 PMC3459158

[6] C. L. Shepard and K. H. Kraft , “The Nonpalpable Testis: A Narrative Review,” Journal of Urology 198 (2017): 1410–1417. 28434984 10.1016/j.juro.2017.04.079 PMC5650944

[7] I. Hamano , S. Hatakeyama , R. Nakamura , et al., “Onco‐Testicular Sperm Extraction (Onco‐TESE) From a Single Testis With Metachronous Bilateral Testicular Cancer: A Case Report,” Basic and Clinical Andrology 28 (2018): 1. 29416867 10.1186/s12610-018-0066-2 PMC5785797

[8] S. Tsutsumi , T. Kawahara , T. Takeshima , et al., “Onco‐Testicular Sperm Extraction (Onco‐TESE) for Bilateral Testicular Tumors: Two Case Reports,” Journal of Medical Case Reports 11 (2017): 139. 28511670 10.1186/s13256-017-1303-6 PMC5434529

[9] K. Hayashi , T. Inamoto , H. Azuma , H. Masuda , and H. Oku , “A Case of Congenital Single Testis With Testicular Cancer Patient and Azoospermia Who Was Able to Collect Spermatozoa With Ipsilateral Onco‐TESE,” Clinical Case Reports 9 (2021): 535–539. 33489210 10.1002/ccr3.3576 PMC7813069

[10] J. Karibe , T. Takeshima , S. Kuroda , et al., “Testicular Sperm Extraction for Fertility Preservation in Young Patients With Cancer,” Translational Andrology and Urology 13 (2024): 1463–1471. 39280651 10.21037/tau-24-21 PMC11399058

[11] K. Furuhashi , T. Ishikawa , H. Hashimoto , et al., “Onco‐Testicular Sperm Extraction: Testicular Sperm Extraction in Azoospermic and Very Severely Oligozoospermic Cancer Patients,” Andrologia 45 (2013): 107–110. 22690948 10.1111/j.1439-0272.2012.01319.x

[12] L. Cirigliano , M. Falcone , M. Gül , et al., “Onco‐TESE (Testicular Sperm Extraction): Insights From a Tertiary Center and Comprehensive Literature Analysis,” Medicina (Kaunas, Lithuania) 59 (2023): 1226. 37512038 10.3390/medicina59071226 PMC10386487

[13] J. M. Flores , I. Henriquez , J. S. Jue , et al., “The Outcomes of Onco‐Testis Sperm Extraction at the Time of Radical Orchiectomy,” Urology 199 (2025): 90–94. 39674379 10.1016/j.urology.2024.12.005 PMC12857814

[14] J. B. Fanshawe , T. Hughes , K. Briggs , et al., “Oncological Microdissection Testicular Sperm Extraction (Onco‐microTESE) Outcomes for Fertility Preservation of Patients With Testicular Cancer With Azoospermia or Severe Oligoasthenoteratozoospermia,” BJU International 135 (2025): 295–302. 39548846 10.1111/bju.16553 PMC11746000

[15] H. C. Lin , W. H. Tang , Y. Chen , Y. Y. Fang , and K. Hong , “Microdissection Testicular Sperm Extraction for Men With Nonobstructive Azoospermia Who Have a Testicular Tumor In Situ at the Time of Sperm Retrieval,” Asian Journal of Andrology 27 (2025): 423–427. 40019188 10.4103/aja2024109 PMC12112931

[16] A. Agarwal and S. S. Allamaneni , “Disruption of Spermatogenesis by the Cancer Disease Process,” Journal of the National Cancer Institute Monographs 34 (2005): 9–12. 10.1093/jncimonographs/lgi005 15784813

[17] J. A. Moody , K. Ahmed , C. Horsfield , M. R. V. Pedersen , T. Yap , and M. Shabbir , “Fertility Preservation in Testicular Cancer ‐ Predictors of Spermatogenesis,” BJU International 122 (2018): 236–242. 29667332 10.1111/bju.14214

[18] B. W. De Jong , C. A. De Gouveia Brazao , H. Stoop , et al., “Raman Spectroscopic Analysis Identifies Testicular Microlithiasis as Intratubular Hydroxyapatite,” Journal of Urology 171 (2004): 92–96. 14665852 10.1097/01.ju.0000101948.98175.94

[19] T. Wang , L. Liu , J. Luo , T. Liu , and A. Wei , “A Meta‐Analysis of the Relationship Between Testicular Microlithiasis and Incidence of Testicular Cancer,” Urology Journal 12 (2015): 2057–2064. 25923148

[20] H. G. Wilson , B. R. Birch , and R. W. Rees , “Is Testicular Microlithiasis Associated With Decreased Semen Parameters? A Systematic Review,” Basic and Clinical Andrology 34 (2024): 23. 39633271 10.1186/s12610-024-00238-x PMC11619182

